# The intersection of rurality and dementia prevalence in Australia for Aboriginal and Torres Strait Islander and non‐Indigenous peoples

**DOI:** 10.5694/mja2.52657

**Published:** 2025-05-02

**Authors:** Antonia J Clarke, Marwan Ahmed, Judith M Katzenellenbogen, John Towney (Wiradjuri), Anna H Balabanski, Adrienne Withall (Dharawal Yuin), Kylie Radford, Amy Brodtmann

**Affiliations:** ^1^ Monash University Melbourne VIC; ^2^ University of Sydney Sydney NSW; ^3^ Cardiovascular Epidemiology Research Centre University of Western Australia Perth WA; ^4^ University of Melbourne Melbourne VIC; ^5^ UNSW Sydney Sydney NSW; ^6^ Neuroscience Research Australia Sydney NSW

**Keywords:** Epidemiology, Indigenous health, Dementia, Rural health services

## Abstract

**Objectives:**

To determine the nationwide prevalence of dementia as it intersects with rurality for Aboriginal and Torres Strait Islander and non‐Indigenous peoples in Australia.

**Study design:**

Cross‐sectional population‐based prevalence study using nationwide respondent‐reported data.

**Setting, participants:**

All people aged 45 years and older, including people living in residential aged care facilities, hospitals and prisons, who responded to the 2021 Australian census.

**Main outcome measures:**

Crude, age‐specific and age‐standardised dementia prevalence, and odds of dementia, for Aboriginal and Torres Strait Islander and non‐Indigenous peoples across remoteness levels.

**Results:**

For non‐Indigenous peoples, the crude and age‐standardised (World Health Organization standard) prevalence of dementia was 10.6 and 7.4 per 1000 persons, respectively. For Aboriginal and Torres Strait Islander peoples, the crude and age‐standardised prevalence was 13.4 and 16.2 per 1000 persons, respectively. The age‐specific prevalence ratio for Aboriginal and Torres Strait Islander and non‐Indigenous peoples was most pronounced at younger age bands (3.5 for 45–49 years, 3.8 for 50–54 years and 3.4 for 60–64 years), narrowing to 1.6 among those aged 80–84 years. Odds of dementia decreased significantly with increasing remoteness for non‐Indigenous peoples, but not for the Aboriginal and Torres Strait Islander population. Increasing age and no educational attainment were strongly associated with increased odds of dementia across both populations.

**Conclusions:**

Consideration of geography is of crucial significance in dementia epidemiology, particularly for Aboriginal and Torres Strait Islander peoples. Our findings confirm those of previous community cohort and linked data studies that highlight the disproportionate burden of dementia borne by Aboriginal and Torres Strait Islander peoples using a national dataset. Place should inform targeted health care policy to address risk and protective factors for dementia prevention and care.

**The known:** Aboriginal and Torres Strait Islander peoples experience greater dementia prevalence at a younger age than non‐Indigenous Australians, yet little is known about urban–rural influences on pathological brain ageing.

**The new:** Using a national dataset, we show that Aboriginal and Torres Strait Islander peoples living in regional and remote Australia are equally or more likely to have a dementia diagnosis compared with their urban counterparts; they have a distinctly different pattern of dementia epidemiology than that observed for non‐Indigenous Australians.

**The implications:** Place should inform dementia awareness strategies and health care policy to direct targeted dementia prevention and care to Aboriginal and Torres Strait Islander peoples.

Dementia is a major cause of death and disability worldwide. In Australia, dementia is already the leading cause of death for women^1^ and cases are expected to double by 2058.^2^ Risk factors for dementia reflect the cumulative life course contribution of biomedical, lifestyle and socio‐economic influences. Health inequity experienced by Aboriginal and Torres Strait Islander peoples in Australia stemming from colonisation and structural racism contributes to an increased risk of neurodegenerative disease.^3^, ^4^ Dementia diagnosis and care is confounded by marginalisation within health care systems and limited service availability, particularly in rural and remote areas.^5^, ^6^ With projected rapid ageing of the Aboriginal and Torres Strait Islander population, dementia is anticipated to contribute significantly to the disease burden in this population.^7^ However, little is known about urban–rural influences on pathological brain ageing for Aboriginal and Torres Strait Islander peoples.

Place matters to health care outcomes, including dementia.^5^, ^6^ Dementia prevalence estimates in rural Australia vary.^8^, ^9^ For Aboriginal and Torres Strait Islander peoples, the cultural significance of connection to Country (ancestral lands, waterways and skies) is a vital consideration in the provision of culturally safe dementia prevention measures and dementia care.^5^, ^10^ Age‐standardised estimates of dementia prevalence for Aboriginal and Torres Strait Islander peoples range from 2.9‐fold^11^ to 5.2‐fold^12^ greater than estimates for the non‐Indigenous population. Researchers have shown that there is no clear trend toward increasing dementia prevalence when comparing urban Australian data with regional data,^13^ nor when comparing urban data with remote data;^12^ however, to our knowledge, no study has directly examined such relationships.

Adequately capturing data to determine dementia prevalence is challenging. Without readily available biomarkers, the diagnosis of dementia is ultimately clinical. Population‐based studies of dementia epidemiology are beset by changing diagnostic criteria and tools, and varying levels of health care professional and public understanding of the disease.^14^, ^15^, ^16^ In 2021, the Australian Bureau of Statistics (ABS) census asked respondents, including those in hospital or residential aged care facilities, whether a doctor or nurse had advised them of a diagnosis of dementia. The census has near‐complete population coverage. Authors of previous studies have demonstrated reasonable concordance between proxy measures of dementia and clinical diagnosis.^17^

By accessing the publicly available dementia data collected in the 2021 census, we aimed to examine the nationwide prevalence of dementia and the intersection with rurality for Aboriginal and Torres Strait Islander and non‐Indigenous peoples.

## Methods

### Data sources

The ABS census is a nationwide survey of all Australians, conducted every 5 years. In 2021, data were collected online, via mail and in person. The census provides a comprehensive health, economic, social and cultural picture of Australia.

### Case definition

In 2021, census respondents were asked to nominate long term health conditions from a pre‐defined list, including whether they had been told by a doctor or nurse that they had a diagnosis of dementia (including Alzheimer disease). People aged 45 years and older who responded to this question were included in our study. The cohort was age‐restricted owing to the low numbers of Aboriginal and Torres Strait Islander peoples aged over 84 years, particularly in more rural areas. Aggregated data pertaining to numbers of dementia cases were obtained from ABS data cubes^18^ and cross‐tabulated by usual residence according to the Australian Statistical Geography Standard – Remoteness Area (ASGS‐RA) classification, Indigenous status, 5‐year age group, sex and education level.

The ASGS‐RA structure divides Australia into five classes of remoteness (major cities, inner regional, outer regional, remote, very remote). Remoteness Areas are based on the Accessibility/Remoteness Index of Australia Plus (ARIA+), produced by the Australian Centre for Housing Research.^19^ Education levels were determined using the level of highest educational attainment variable, which records a single measure of a person's educational attainment. Education levels were stratified into no educational attainment (no school or non‐school qualification), Year 9 or below (9 or fewer years of formal education), Year 10 or above (10 or more years of formal education), postgraduate qualification (tertiary education), and not stated or inadequately described.

### Data analysis

Dementia prevalence was calculated for Aboriginal and Torres Strait Islander and non‐Indigenous populations using 5‐year age groups. Crude overall and age‐specific estimates were obtained using the number of affirmative responses to the dementia question (numerator) and the number of people included in the census (denominator). Participants who responded “not stated” to the dementia question were included in the denominator to provide a conservative estimate of dementia prevalence. A sensitivity analysis, which excluded these responses from the denominator, was used to assess whether this approach provided biased estimates.

Age‐standardised prevalence for Aboriginal and Torres Strait Islander and non‐Indigenous populations aged 45–84 years was calculated using the direct method. Two standard population distributions, the 2021 Australian population and the World Health Organization (WHO) standard, were used to calculate age‐standardised prevalence to enable comparison with other studies. Logistic regression modelling was applied to non‐Indigenous and Aboriginal and Torres Strait Islander populations separately to determine predictors of dementia, including age, sex, educational attainment and remoteness level. Logistic regression modelling was restricted to those aged 45–74 years owing to the low number of Aboriginal and Torres Strait Islander respondents with dementia who were aged over 75 years, especially in more remote areas. Statistical analysis was performed using Excel version 16 (Microsoft) and Stata statistical software version 18 (StataCorp).

### Ethical considerations and Indigenous governance

The conceptualisation and design of this study and interpretation of our findings were co‐led and governed by Aboriginal and Torres Strait Islander clinicians, researchers and community members. Informed by Aboriginal cultural and social worldviews, co‐authors John Towney, a proud Wiradjuri man, and Adrienne Withall, a proud Dharawal Yuin and Anglo‐Celtic woman, provided critical input throughout the study process, including writing and editing this article. The study engaged an Aboriginal reference group — composed of lived experience members, health care workers, community Elders and researchers from across Jagera, Gumbaynggirr, Biripi, Malyangapa, Wiradjuri, Wailwan and Kamilaroi nations — to prioritise the advancement of health outcomes for Aboriginal and Torres Strait Islander peoples. Full positionality statements from all authors are included in Box 1. Ethics approval for the project was granted by the Monash University Human Research Ethics Committee (2023/38647) and the Aboriginal Health and Medical Research Council of NSW (2141/23). The Consolidated Criteria for Strengthening the Reporting of Health Research Involving Indigenous Peoples (CONSIDER) checklist was employed to guide strengths‐based reporting of research involving Indigenous peoples (Supporting Information, table 1).^20^

Box 1 **Author positionality statements**

**Table:** 

I, Antonia Clarke, am a New Zealander by birth and have lived on Gadigal land for most of my adult life. I am currently living and working on the lands of the Wurundjeri peoples of the Kulin nation. I am a wife and mother of three, a neurologist, and a researcher with a deep interest in health equity and lifelong brain health. I trained as a doctor across rural and remote New South Wales following a previous career in law. Combined, these experiences granted me a tangible appreciation of Aboriginal wellbeing that is inclusive of family, Culture and Community.
I, Marwan Ahmed, am Sudanese by birth and have lived, studied and worked on the lands of the Whadjuk people of the Noongar nation for the past 5 years. I am a father of three. My PhD research, which explored the impact of diabetes during pregnancy on Aboriginal babies, provided me with the opportunity to build meaningful relationships with Aboriginal communities and gain valuable insights into Aboriginal health. This experience has deepened my understanding and commitment to contributing to the improvement of Aboriginal health outcomes.
I, Judith Katzenellenbogen, am a non‐Aboriginal epidemiologist working on Noongar Country in Western Australia. I have broad public health experience, including in linked data and mixed methods approaches to the study of chronic diseases, particularly focused on Aboriginal cardiovascular health. I have a strong commitment to capacity building, research partnerships and research translation that can support Aboriginal health and wellbeing.
I, John Towney, am a proud Wiradjuri man from the Central Western NSW town of Wellington. My grandfather is from the Bulgandramine Mission near Peak Hill in Western NSW. My great grandfather was William “Wondong” Towney, a proud descendant of the Bogan River Wiradjuri, and my great grandmother was Elizabeth “Lizzie” Merritt, a Wiradjuri midwife of the Bogan River Country. I spent my younger years learning about and connecting to my family, Community and ancestors. This has led me on a lifelong journey of knowing in the Wiradjuri sense and sharing what I have known with those around me who are working to change the connections between Aboriginal and non‐Aboriginal peoples. I commenced my journey into tertiary education at a relatively late age of 45 years and obtained my Bachelor of Medicine at age 50 years. In the 8 years since, I have consistently worked on the translation of understanding the Aboriginal ways of knowing, being and doing in the research context, to the practical context of relating and therefore implementing those research findings into practice.
I, Anna Balabanski, am of European descent and currently live on the lands of the Wurundjeri people. I have a longstanding commitment to Aboriginal health and community, having had a strong personal connection to Aboriginal people since childhood, including connections with the Papunya Aboriginal community in the Northern Territory. I have also had a close connection to Aboriginal Culture through my medical practice, including working as a visiting neurologist at Alice Springs Hospital. I acknowledge my privilege, as someone of European heritage being offered the opportunity to work together with Aboriginal researchers and communities towards equity in health care across Australia. I am committed to ongoing learning about inclusivity of family, Community and Culture within Aboriginal health care in research and clinical practice.
I, Adrienne Withall, am a Dharawal Yuin and Anglo‐Celtic woman. I was raised, and am fortunate to still live, surrounded by beautiful Bidjigal lands and waters. I am a mother and wife, an academic, and an associate professor in the School of Psychology at UNSW Sydney. My research is centred upon amplifying the voice of people ageing at the margins, which includes Aboriginal and Torres Strait Islander peoples, and identifying and addressing equity and social justice issues in dementia research. I am connected to and give back to my community through my involvement in the NSW Aboriginal Education Consultative Group, NSW Ministerial Advisory Council on Ageing, International Indigenous Dementia Research Network, and OCHRe (Our Collaborations in Health Research) Network of Aboriginal and Torres Strait Islander researchers.
I, Kylie Radford, am a woman of Anglo‐Celtic descent, raised on Wiradjuri land and now living and working on Bidjigal and Gadigal lands. I am a wife and mother, clinical neuropsychologist, conjoint associate professor in the School of Psychology at UNSW Sydney, and senior research scientist at Neuroscience Research Australia, where I have spent 15 years with the Aboriginal Health and Ageing Program, including leading research to understand dementia in partnership with Aboriginal communities across NSW.
I, Amy Brodtmann, am Australian by birth with mixed German, Norwegian, British and Chinese genetic heritage. I was born on the lands of the Brayakaulung people of the Gunaikurnai nation and I have lived on the lands of the Wurundjeri Woi‐Wurrung people of the Kulin nation for most of my adult life. I am the youngest of three daughters, raised by a single mother, and I am a mother of three daughters. I am a professor of neuroscience, a neurologist, and a researcher whose vision and mission are brain health for all. My life experience allows me to have a deep respect and recognition of Aboriginal wellbeing that is inclusive of family, Culture and Community.

## Results

### Demographic profile of the population as a whole

In 2021, 9 921 701 of 24 701 703 non‐Indigenous peoples (40.2%) were aged over 45 years, compared with 187 952 of 812 728 Aboriginal and Torres Strait Islander peoples (23.1%) (Box 2). In the 45–84‐year age category, a higher proportion of non‐Indigenous (8 471 974; 89.9%) and Aboriginal and Torres Strait Islander (119 663; 64.5%) peoples lived in major cities or inner regional areas than in more remotes areas (Box 3). The relative proportion of the population identifying as Aboriginal and Torres Strait Islander, however, increased with remoteness. While there were similar proportions of Aboriginal and Torres Strait Islander males across remoteness levels, non‐Indigenous males comprised a higher proportion of the very remote population (20 450; 55.7%) compared with the major cities population (3 130 058; 47.8%). The proportion of Aboriginal and Torres Strait Islander peoples with educational attainment of Year 10 or above was lower in very remote areas (9423; 54.7%) compared with major cities (52 984; 73.6%) whereas this remained constant across remoteness for non‐Indigenous peoples.

Box 2 **Numbers and percentages of 2021 census respondents aged ≥ 45 years and 45–84 years, and number in each group who self‐reported having received a diagnosis of dementia, by Indigenous status**

**Table:** 

	Non‐Indigenous	Aboriginal and Torres Strait Islander
Total respondents	24 701 703	812 728
People aged ≥ 45 years, *n* (%)	9 921 701 (40.2%)	187 952 (23.1%)
People with dementia, *n*	179 652	2971
People aged 45–84 years, *n* (%)	9 421 506 (38.1%)	185 612 (22.8%)
People with dementia, *n*	99 501	2492

Box 3 **Numbers and percentages of 2021 census respondents aged 45–84 years who were male and had Year 10 or above education, by Indigenous status and remoteness level**

**Table:** 

	Remoteness level				
	Major cities	Inner regional	Outer regional	Remote	Very remote
**Non‐Indigenous (*n* = 9 421 506)**					
Respondents, *n* (%)*	6 543 095 (69.5%)	1 928 879 (20.5%)	822 619 (8.7%)	90 169 (1.0%)	36 744 (0.4%)
Men, *n* (%)^†^	3 130 058 (47.8%)	933 369 (48.4%)	412 093 (50.1%)	47 930 (53.2%)	20 450 (55.7%)
Year 10 or above, *n* (%)^†^	5 431 410 (83.0%)	1 561 527 (81.0%)	655 831 (79.7%)	73 543 (81.6%)	29 606 (80.6%)
**Aboriginal and Torres Strait Islander (*n* = 185 612)**					
Respondents, *n* (%)*	71 985 (38.8%)	47 678 (25.7%)	37 629 (20.3%)	11 092 (6.0%)	17 228 (9.3%)
Men, *n* (%)^†^	33 579 (46.7%)	22 642 (47.5%)	17 729 (47.1%)	5178 (46.7%)	8036 (46.6%)
Year 10 or above, *n* (%)^†^	52 984 (73.6%)	32 900 (69.0%)	24 961 (66.3%)	6776 (61.1%)	9423 (54.7%)

* Percentages of respondents are row percentages. † Percentages of men and those with Year 10 or above education are column percentages.

### Dementia prevalence

In the non‐Indigenous population aged 45–84 years, 99 501 dementia cases were identified (Box 2), representing a crude prevalence of 10.6 per 1000 persons (Supporting Information, table 2). In the Aboriginal and Torres Strait Islander population, 2492 dementia cases were identified (Box 2), representing a crude prevalence of 13.4 per 1000 persons (Supporting Information, table 2). The age‐specific prevalence ratio for Aboriginal and Torres Strait Islander and non‐Indigenous peoples was most pronounced at younger age bands (3.5 for 45–49 years, 3.8 for 50–54 years and 3.4 for 60–64 years) with the ratio narrowing to 1.6 in the 80–84‐year age band (Box 4).

Box 4 Age‐specific dementia prevalence and prevalence ratios for Aboriginal and Torres Strait Islander and non‐Indigenous peoples aged 45–84 years, 2021*

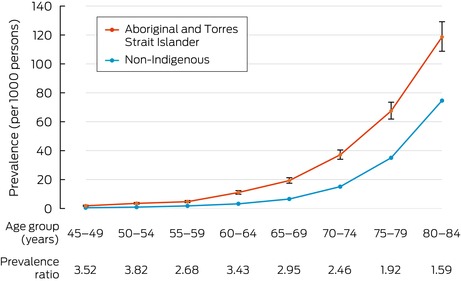

* Error bars represent 95% CIs.

Using the WHO standard population for 45–84 years, age‐standardised dementia prevalence was 7.4 per 1000 persons for the non‐Indigenous population and 16.2 per 1000 persons for the Aboriginal and Torres Strait Islander population, with an age‐standardised prevalence ratio of 2.2 (Box 5). Employing the 2021 Australian population, overall prevalence was 10.5 per 1000 persons for the non‐Indigenous population and 22.0 per 1000 persons for the Aboriginal and Torres Strait Islander population, with a prevalence ratio of 2.1. The prevalence ratio increased with remoteness level, regardless of the standard population employed. There was no significant sex difference in age‐standardised prevalence for the Aboriginal and Torres Strait Islander population. Exclusion of “not stated” responses from the denominator did not substantially alter prevalence estimates.

Box 5 **Directly age‐standardised dementia prevalence (per 1000 persons) and prevalence ratio, by Indigenous status and remoteness level for people aged 45–84 years, employing the WHO standard and Australian 2021 standard**

**Table:** 

	Age‐standardised prevalence for non‐Indigenous people		Age‐standardised prevalence for Aboriginal and Torres Strait Islander people		Aboriginal and Torres Strait Islander to non‐Indigenous prevalence ratio	
	WHO standard (95% CI)	Australian 2021 standard (95% CI)	WHO standard (95% CI)	Australian 2021 standard (95% CI)	WHO standard	Australian 2021 standard
All respondents aged 45–84 years	7.4 (7.4–7.5)	10.5 (10.4–10.6)	16.2 (15.6–16.8)	22.0 (21.1–22.8)	2.2 (2.2–2.2)	2.1 (2.1–2.1)
Sex						
Male	7.6 (7.5–7.6)	10.6 (10.5–10.7)	16.2 (15.2–17.1)	21.4 (20.1–22.7)	2.1 (2.1–2.2)	2.0 (2.0–2.1)
Female	7.2 (7.2–7.3)	10.4 (10.3–10.5)	16.2 (15.3–17.1)	22.4 (21.2–23.6)	2.2 (2.2–2.3)	2.2 (2.1–2.2)
Remoteness level						
Major cities	7.6 (7.5–7.7)	10.8 (10.7–10.9)	15.2 (14.2–16.2)	20.7 (19.3–22.1)	2.0 (1.7–2.3)	1.9 (1.7–2.2)
Inner regional	7.2 (7.1–7.3)	10.1 (9.9–10.2)	15.4 (14.3–16.6)	20.5 (18.9–22.1)	2.2 (1.7–2.7)	2.0 (1.7–2.5)
Outer regional	6.9 (6.7–7.1)	9.7 (9.5–9.9)	16.7 (15.3–18.1)	22.6 (20.6–24.5)	2.4 (1.7–3.4)	2.3 (1.7–3.1)
Remote	5.9 (5.4–6.3)	8.3 (7.7–9.0)	21.1 (18.1–24.2)	30.2 (25.7–34.7)	3.6 (2.5–5.3)	3.6 (2.6–5.0)
Very remote	5.3 (4.6–6.0)	7.8 (6.7–8.8)	18.8 (16.4–21.1)	25.4 (22.0–28.8)	3.6 (2.0–6.5)	3.3 (1.9–5.5)

WHO = World Health Organization.

### Adjusted odds of dementia

Age and educational attainment significantly influenced the odds of dementia for people aged 45–74 years in both Aboriginal and Torres Strait Islander and non‐Indigenous populations (Box 6). Age had the strongest positive association for both groups based on adjusted odds ratios (aORs); relative to the 45–49‐year group, odds of dementia increased as age increased.

Box 6 **Adjusted odds ratios for dementia for people aged 45–74 years, by Indigenous status**

**Table:** 

	Adjusted odds ratio (95% CI)	
	Non‐Indigenous	Aboriginal and Torres Strait Islander
Age group, years		
45–49	1.0	1.0
50–54	1.7 (1.6–1.9)*	1.9 (1.4–2.5)*
55–59	3.1 (2.9–3.4)*	2.4 (1.8–3.2)*
60–64	5.6 (5.2–6.0)*	5.4 (4.2–7.0)*
65–69	11.0 (10.2–11.8)*	8.9 (6.9–11.4)*
70–74	23.7 (22.1–25.5)*	15.8 (12.3–20.2)*
Sex		
Female	0.8 (0.8–0.8)*	0.9 (0.8–1.0)
Remoteness level		
Major cities	1.0	1.0
Inner regional	1.0 (1.0–1.0)	1.1 (1.0–1.3)
Outer regional	0.9 (0.9–0.9)*	1.2 (1.0–1.3)*
Remote	0.8 (0.7–0.9)*	1.0 (0.8–1.3)
Very remote	0.5 (0.4–0.7)*	1.0 (0.9–1.3)
Education level		
Postgraduate	0.3 (0.3–0.4)*	0.2 (0.2–0.3)*
Year 10 or above	0.6 (0.6–0.6)*	0.6 (0.5–0.6)*
Year 9 or below	1.0	1.0
No educational attainment	2.2 (2.1–2.3)*	2.3 (1.8–2.8)*
Not stated or inadequately described	1.3 (1.2–1.3)*	1.1 (0.9–1.2)

* Significant association.

Relative to an educational level of Year 9 or below, no educational attainment was significantly associated with increased odds of dementia (aOR, 2.2 [95% CI, 2.1–2.3] for the non‐Indigenous population; aOR, 2.3 [95% CI, 1.8–2.8] for the Aboriginal and Torres Strait Islander population), whereas higher levels of educational attainment (Year 10 education or above) significantly reduced the odds of dementia (aOR, 0.6 [95% CI, 0.6–0.6] for the non‐Indigenous population; aOR, 0.6 [95% CI, 0.5–0.6] for the Aboriginal and Torres Strait Islander population).

Relative to residents of major cities, the odds of dementia significantly decreased with each level of increasing remoteness for the non‐Indigenous population (aOR, 0.5 [95% CI, 0.4–0.7] for very remote residents). By contrast, for the Aboriginal and Torres Strait Islander population, the odds of dementia were significantly increased for outer regional areas (aOR, 1.2; 95% CI, 1.0–1.3), with results for inner regional, remote and very remote areas not significantly different to those for major cities.

Sex had no significant effect on the odds of dementia for Aboriginal and Torres Strait Islander peoples, whereas odds of dementia were lower for women across the non‐Indigenous population aged 45–74 years. When the age range was increased to 45–99 years, the odds of dementia were significantly greater for non‐Indigenous women (aOR, 1.0; 95% CI, 1.0–1.0).

## Discussion

Our findings show that there are distinctly different patterns of dementia epidemiology for Aboriginal and Torres Strait Islander and non‐Indigenous peoples according to geography. Dementia prevalence increased for Aboriginal and Torres Strait Islander peoples with greater remoteness and, conversely, decreased for non‐Indigenous Australians. As such, the more than twofold increase in the overall age‐standardised prevalence ratio for Aboriginal and Torres Strait Islander peoples compared with the non‐Indigenous population widened to more than 3.5‐fold in remote and very remote regions. When controlling for variation in age, sex and education levels, the odds of dementia remained constant or increased with remoteness for Aboriginal and Torres Strait Islander peoples, but decreased for the non‐Indigenous population. These findings have major implications for considerations of dementia aetiology, risk factors, diagnosis and care for Aboriginal and Torres Strait Islander peoples across urban to remote settings.

Globally, the prevalence of dementia is purported to be greater in rural compared with urban areas.^21^ The disproportionate representation of older peoples in more rural areas due to rural–urban migration may underlie this finding.^6^ The rural–urban gap in access to primary care and specialist services, and the increased prevalence of known risk factors for dementia (including cardiometabolic disease), are also likely to contribute.^22^, ^23^

It is important to contextualise health‐related outcomes within the complexity of rural environments.^6^ Australia is characterised by vast and challenging landscapes, and many older Australians seek proximity to health care services pooled in more accessible areas, contributing to lower national dementia prevalence estimates in rural areas.^8^, ^24^ For Aboriginal and Torres Strait Islander peoples, a more holistic view of health that incorporates social and emotional wellbeing may influence health‐seeking behaviour.^25^ Connection to Country and proximity to family and community are forefront considerations. When faced with a diagnosis of dementia, many will prioritise culturally appropriate and community‐based approaches to care,^26^ including remaining on or returning to Country.^5^ Our age‐adjusted results support this premise and previous findings that describe differing trends for dementia‐related mortality between Aboriginal and Torres Strait Islander and non‐Indigenous populations according to geography.^27^

To our knowledge, our study is the first national snapshot of dementia prevalence for Aboriginal and Torres Strait Islander and non‐Indigenous Australians that has examined data from the 2021 census. The census offers almost complete population coverage, with inclusion of aged care facility residents, diverse community groups, and people living in remote areas. The crude estimates were lower than those previously identified from another national dataset.^28^ The lower overall prevalence may indicate an active evolution of the disease, yet data relating to incidence trends across Indigenous populations are limited.^29^, ^30^

We found that the crude dementia prevalence for Aboriginal and Torres Strait Islander peoples aged 45–84 years was 1.3 times that for the non‐Indigenous population. When age‐standardised to the WHO population, the prevalence ratio increased to 2.2. These estimates are lower than those seen in most previous community cohort and linked data studies, with varying age bands, methods and standard populations employed, and modelling for dementia under‐diagnosis limiting direct comparison.^11^, ^12^, ^13^, ^31^ Our findings are most in keeping with those of a study that used linked hospital and mortality data for all age groups in New South Wales, which showed an age‐adjusted dementia prevalence ratio of 2.02 (95% CI, 1.80–2.27).^32^

Age‐specific prevalence trends may identify the subgroups at higher risk of dementia more accurately than overall trends. We confirmed greater dementia prevalence ratios at younger age bands for the Aboriginal and Torres Strait Islander population, with more precise age‐specific estimates possible compared with previous studies.^11^, ^12^, ^31^ The younger age at diagnosis may reflect the earlier accumulation of known dementia risk factors in the Aboriginal and Torres Strait Islander population, as well as resilient survival of older adults in this population. Future research using individual‐level data is required to examine the influence of other modifiable contributors to the disease.

Our study also supports educational attainment as an important modifier of dementia risk for both populations. Higher levels of education had a significant protective effect in terms of dementia outcomes, irrespective of age, sex or geography. This finding endorses current Closing the Gap targets regarding high school attainment, with a Year 10 or lower education accounting for 9.5% of dementia cases among Aboriginal and Torres Strait Islander peoples in a recent analysis^4^ — more than any other modifiable risk factor examined. The data obtained from the census did not provide scope to consider other forms of cognitive engagement in early‐to‐mid life, particularly those that may be more applicable to Aboriginal and Torres Strait Islander peoples^3^ and those that may address issues of educational access for older generations, especially women.

Finally, we showed that sex did not significantly alter the odds of dementia for the Aboriginal and Torres Strait Islander population, distinct from the findings of some previous studies.^12^, ^33^ Interestingly, given the generally higher prevalence of dementia in women, we found a higher age‐standardised prevalence of dementia for non‐Indigenous men in the younger age category of 45–84 years. When age bands up to 99 years were employed in logistic regression modelling, female sex lost its protective effect.

### Limitations

Limitations of our study include the method of case ascertainment in the census (receipt of a dementia diagnosis). Dementia is universally underdetected and under‐reported across community and health care settings,^16^ and more so for Aboriginal and Torres Strait Islander peoples for whom societal, cultural and linguistic barriers entrench limited access to and use of services.^31^, ^34^ Thus, dementia prevalence for Aboriginal and Torres Strait Islander peoples is probably underestimated, especially in more remote areas. Future validation of dementia response data is required to clarify both numerator and denominator bias.^35^, ^36^ Also, we assumed adequate identification of Indigenous status in the census. The census relies on self‐identification, and the non‐response rate for Indigenous status was 4.9% in 2021.^37^ In addition, we acknowledge that the use of the Australian population and, to a lesser extent, the WHO standard to age standardise may reduce the magnitude of inequities and width of confidence intervals relative to standardising to the (generally younger) age structure of Indigenous populations.^38^, ^39^, ^40^

### Conclusions

The inclusion of dementia as a chronic health condition in the 2021 census is evidence of its political, economic and social importance in Australia's future. Our results, which drew on a comprehensive national dataset, emphasise that geographic considerations are of crucial significance in dementia epidemiology, particularly for Aboriginal and Torres Strait Islander peoples. Our findings also confirm the disproportionate burden of dementia borne by Aboriginal and Torres Strait Islander peoples at a younger age. Our results should inform the development of targeted, place‐based educational strategies and culturally safe, consultative health care policy to address dementia risk, protective factors and care.

## Open access

Open access publishing facilitated by Monash University, as part of the Wiley ‐ Monash University agreement via the Council of Australian University Librarians.

## Competing interests

No relevant disclosures.

## Data sharing

All authors had full access to the data related to this study. The original data can be accessed from the Australian Bureau of Statistics. The study data can be accessed by contacting the corresponding author.

## Supporting information


Supplementary tables
