# Supplementary material for: The intersection of rurality and dementia prevalence in Australia for Aboriginal and Torres Strait Islander and non‐Indigenous peoples
Source: Med J Aust. 2025 May 2;222(10):510–6. doi: 10.5694/mja2.52657 (PMC12126967; doi:10.5694/mja2.52657)
Supplement: Supplementary file 1 — Supplementary tables [file MJA2-222-510-s001.pdf]

The Medical Journal of Australia

Supporting Information

Supplementary material

**This appendix was part of the submitted manuscript and has been peer reviewed.
It is posted as supplied by the authors.**

Appendix to: Clarke AJ, Ahmed M, Katzenellenbogen JM, et al. The intersection of rurality and dementia prevalence in Australia for Aboriginal and Torres Strait Islander and non-Indigenous peoples. *Med J Aust* 2025; doi: 10.5694/mja2.52657.

Table 1: Demonstrated adherence to the CONSolIDated critERia for strengthening the reporting of health research involving Indigenous Peoples (CONSIDER) checklist domains to support the advancement of equity in Indigenous health and research

Research domain	Study practice
Governance	Letters of support obtained from urban, regional, and rurally based ACCHS to undertake study. Formation of an Aboriginal Reference Group, comprised of lived experience, community members, researchers, and healthcare workers, to oversee data analysis and interpretation.
Prioritisation	Study aim developed in collaboration with Aboriginal and Torres Strait Islander stakeholders (Aboriginal co-authors, Reference Group and ACCHS members).
Relationships	Ethical approval sought from the Aboriginal Health and Medical Research Council. Senior co-authors established in field of Indigenous health and research.
Methodologies	Study recognises the life course influences on pathological brain ageing, including the major impact of colonisation and structural racism for Aboriginal and Torres Strait Islander peoples.
Participation	Preliminary findings presented at a state-level conference to seek feedback from Aboriginal and Torres Strait Islander healthcare workers, researchers and community members on the planned statistical analysis and interpretation of publicly available data.
Capacity	Active recruitment of Aboriginal researchers (as co-authors) to assist in study design and interpretation.
Analysis and interpretation	Multivariate logistic regression modelling applied separately to non-Indigenous and Aboriginal and Torres Strait Islander populations to examine population-specific influence of variables shaping predictors of dementia, rather than comparative modelling.
Dissemination	Preliminary findings presented at a state-level conference to seek feedback and interpretation of results from Aboriginal and Torres Strait Islander healthcare workers, researchers, and community members. Finalised manuscript distributed to study Reference Group and employed in yarning circles with Aboriginal and Torres Strait Islander peoples in regional and remote communities to support local knowledge translation.

ACCHS = Aboriginal Community Controlled Health Service.

Table 2. Crude dementia prevalence (per 1000 persons) and prevalence ratio for Aboriginal and Torres Strait Islander peoples and non-Indigenous peoples aged 45 years and older and 45–84 years, 2021

CRUDE PREVALENCE							
	**Non-Indigenous**			**Aboriginal and Torres Strait Islander**			**Aboriginal and Torres Strait Islander to non-Indigenous prevalence ratio (95% CI)**
	**Cases (persons)**	**Denominator (persons)**	**Prevalence (95% CI)**	**Cases (persons)**	**Denominator (persons)**	**Prevalence (95% CI)**	
**TOTAL POPULATION**							
**45+ years**	**179,672**	**9,921,701**	**18.1 (18.0, 18.2)**	**2,971**	**187,952**	**15.8 (15.3, 16.4)**	**0.9 (0.8, 0.9)**
**45-84 years**	**99,501**	**9,421,506**	**10.6 (10.5, 10.6)**	**2,492**	**185,612**	**13.4 (12.9, 14.0)**	**1.3 (1.2, 1.3)**
**Age-specific (years)**							
45-49	783	1,513,366	0.5 (0.5, 0.6)	75	40,908	1.8 (1.5, 2.3)	3.5 (2.8, 4.5)
50-54	1,373	1,493,425	0.9 (0.9, 1.0)	139	39,562	3.5 (3.0, 4.2)	3.8 (3.2, 4.6)
55-59	2,521	1,433,951	1.8 (1.7, 1.8)	157	33,314	4.7 (4.0, 5.5)	2.7 (2.3, 3.2)
60-64	4,416	1,372,627	3.2 (3.1, 3.3)	305	27,568	11.1 (9.9, 12.4)	3.4 (3.1, 3.9)
65-69	7,983	1,219,018	6.6 (6.4, 6.7)	386	20,010	19.3 (17.5, 21.3)	3.0 (2.7, 3.3)
70-74	16,511	1,094,622	15.1 (14.9, 15.3)	493	13,279	37.1 (34.0, 40.5)	2.5 (2.3, 2.7)
75-79	27,148	774,835	35.0 (34.6, 35.5)	480	7,117	67.4 (61.9, 73.5)	1.9 (1.8, 2.1)
80-84	38,766	519,662	74.6 (73.9, 75.3)	457	3,854	118.6 (108.7, 129.2)	1.6 (1.4, 1.7)

CI = confidence interval.
